# Longitudinal Associations Between Early Physical Literacy and Physical Activity During Adolescence: A Narrative Review

**DOI:** 10.3390/children13091249

**Published:** 2026-09-15

**Authors:** Sarah A. Costigan, Carl D. Ridgeway, Samantha Vlcek

**Affiliations:** School of Education, College of Design and Social Context, RMIT University, Melbourne, VIC 3000, Australia; carl.ridgeway@rmit.edu.au (C.D.R.); samantha.vlcek@rmit.edu.au (S.V.)

**Keywords:** physical literacy, motor competence, physical activity, adolescence, longitudinal associations

## Abstract

**Highlights:**

**What are the main findings?**

- Childhood motor competence shows the most consistent longitudinal association with physical activity during adolescence.
- Evidence linking multidimensional physical literacy in childhood with later physical activity remains limited and heterogeneous.

**What are the implications of the main findings?**

- Motor competence may represent an important component of childhood physical literacy for supporting continued physical activity into adolescence.
- Further longitudinal research using multidimensional physical literacy measures and objective physical activity assessment is needed to clarify developmental associations and potential pathways.

**Abstract:**

Introduction: Physical literacy is increasingly recognized as a multidimensional construct that may contribute to lifelong physical activity (PA) and health. Understanding how childhood physical literacy relates to PA during adolescence is important given the well-documented decline in adolescent PA worldwide. This narrative review aimed to: (a) identify and critically appraise longitudinal studies examining associations between childhood physical literacy, or its constituent domains, and adolescent PA; (b) explore potential mechanisms and contextual factors influencing these relationships; and (c) identify gaps to inform future research, policy, and practice. Methods: A literature search was conducted across PubMed, Web of Science, SPORTDiscus, Scopus, and PsycINFO for peer-reviewed English-language articles published from 2000 onwards. Eligible primary studies included longitudinal cohort, prospective, or follow-up studies examining childhood physical literacy or recognized domains in relation to adolescent PA. Systematic reviews and meta-analyses were considered as secondary evidence where they contained relevant longitudinal findings. Findings were synthesized narratively. Results: Two primary longitudinal studies met the applied eligibility criteria. These studies examined motor competence and perceived physical competence, respectively, rather than multidimensional physical literacy. Associations with subsequent PA were positive but based on a very small and heterogeneous evidence base. Conclusions: Within the limited set of primary studies identified, motor competence and perceived physical competence showed positive longitudinal associations with subsequent PA; however, the overall evidence remains limited and heterogeneous, and direct longitudinal evidence for multidimensional physical literacy is insufficient. Conclusions should therefore be interpreted cautiously, particularly because no formal risk-of-bias assessment was undertaken.

## 1. Introduction

Physical literacy has emerged as a central concept in promoting lifelong physical activity (PA), health, and well-being. Endorsed by the International Physical Literacy Association (IPLA), physical literacy is defined as “*the motivation, confidence, physical competence, knowledge and understanding to value and take responsibility for engagement in physical activities for life*” [1]. This definition conceptualizes physical literacy as a holistic, multidimensional construct encompassing affective (motivation and confidence), physical (movement competence), cognitive (knowledge and understanding), and behavioral (engagement in PA) domains [2,3,4,5,6]. Rather than functioning independently, these domains interact dynamically throughout the lifespan, shaping an individual’s willingness, capability, and confidence to participate in meaningful movement experiences across educational, recreational, sporting, and everyday contexts. The United Nations Educational, Scientific and Cultural Organization (UNESCO) has recognized physical literacy as an important component of lifelong PA and health, alongside related international policy initiatives [2].

Importantly, physical literacy should be distinguished from related yet more niche constructs, such as physical fitness and motor competence. Physical fitness refers primarily to physiological attributes, while motor competence focuses on proficiency in fundamental movement skills. Although motor competence represents an important component of physical literacy, physical literacy is a broader multidimensional construct encompassing affective, cognitive, physical, and behavioral dimensions, including motivation, confidence, knowledge and understanding, and engagement in physical activity [3,4,5,6]. This distinction is particularly relevant when interpreting the available evidence, as many longitudinal studies have examined motor competence rather than multidimensional physical literacy. Throughout this narrative review, findings relating to motor competence are therefore interpreted as representing one important domain of physical literacy, rather than physical literacy as a whole.

Regular PA during childhood and adolescence is associated with physical, psychosocial and cognitive benefits [7,8], while participation commonly declines during adolescence [9,10]. This decline occurs alongside substantial biological, psychological and social changes, making adolescence an important period for understanding factors associated with sustained PA. The physical literacy framework may provide a useful developmental perspective because competence, confidence, motivation and knowledge may shape young people’s capacity and willingness to remain active [8]. The World Health Organization recommends regular PA throughout childhood and adolescence [11].

Although cross-sectional research generally reports positive associations between physical literacy and PA, cross-sectional designs cannot establish temporal relationships. Longitudinal research is therefore needed to examine whether physical literacy assessed during childhood is associated with subsequent PA during adolescence. This is particularly relevant given the potential contribution of multiple personal, social and environmental factors to PA trajectories [12].

Previous reviews have synthesized associations between physical literacy and PA or have focused on individual domains such as motor competence [3,13,14]. However, previous reviews have not specifically synthesized longitudinal evidence examining childhood multidimensional physical literacy and recognized physical literacy domains in relation to PA during adolescence. The present review therefore focuses specifically on temporal associations while distinguishing evidence relating to multidimensional physical literacy from evidence concerning individual constituent domains. The review also considers the quality, magnitude and limitations of the available evidence. Barutcu et al. further highlight the importance of PA and fitness during childhood and adolescence in relation to longer-term health (metabolic syndrome), reinforcing the public health relevance of understanding factors associated with adolescent PA [15].

Accordingly, the purpose of this narrative review was to synthesize and critically evaluate longitudinal evidence examining the relationship between early physical literacy and PA during adolescence. Specifically, the review aimed to: (a) identify and critically appraise longitudinal studies examining associations between childhood physical literacy, or its constituent domains, and adolescent PA; (b) examine potential mechanisms and contextual factors influencing these relationships; and (c) identify gaps in the evidence to inform future research, policy and practice.

## 2. Methods

### 2.1. Narrative Review Design

This study was conducted as a narrative review to synthesize the emerging longitudinal evidence examining the association between childhood physical literacy and adolescent PA. A narrative review methodology was considered appropriate because of the conceptual and methodological heterogeneity across the available literature, including variation in definitions of physical literacy, measurement approaches, study designs, and outcome measures. A structured literature search, predefined eligibility criteria, and independent screening procedures were implemented to enhance transparency and rigour within the narrative review approach. Primary longitudinal studies were treated as the main evidence base, while systematic reviews and meta-analyses were considered separately as contextual secondary evidence. The quality and reporting of the narrative review were also considered with reference to the Scale for the Assessment of Narrative Review Articles (SANRA) [16].

### 2.2. Literature Search Strategy

A literature search was undertaken to identify studies examining the longitudinal relationship between childhood physical literacy and subsequent PA during adolescence. Electronic searches were conducted across PubMed, Web of Science, SPORTDiscus, Scopus, and PsycINFO to provide coverage of health, sport science, education, psychology and behavioral research.

Search concepts combined terms relating to physical literacy, childhood/adolescence, longitudinal study designs, and PA. The reported concepts were: “physical literac*”; “child*”, “youth”, “adolescent*”, or “teen*”; “longitudinal”, “prospective”, “follow-up”, or “cohort”; “physical activit*”, “movement behaviour*”, or “motor competenc*”; and “tracking”, “trajectory*”, or “development”, combined using Boolean operators. The original database-specific search strings, subject headings, search fields, filters, and exact search dates were not retained. Accordingly, the search cannot be reproduced in full; however, the principal search concepts and databases are reported here to provide transparency regarding the literature identification process.

**Figure 1:**
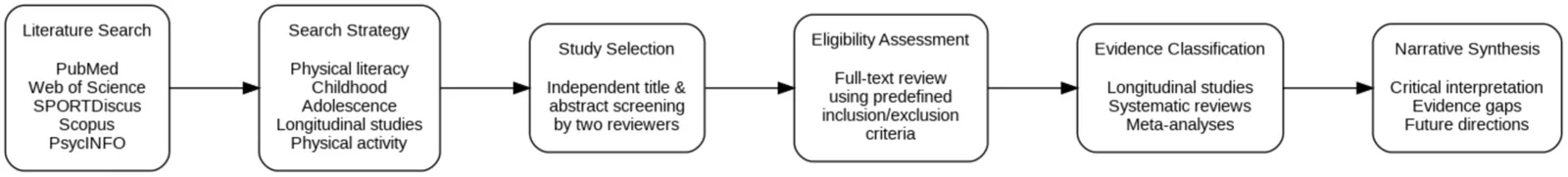
Overview of the narrative review process. A structured literature search was conducted across five electronic databases, followed by independent study selection, eligibility assessment using predefined inclusion and exclusion criteria, classification of evidence, and narrative synthesis.

The search was restricted to peer-reviewed articles published in English from 2000 onwards. Reference lists of eligible studies and relevant review articles were also manually screened to identify additional publications not retrieved through database searches (see Figure 1). The absence of the original database-specific search records is acknowledged as a methodological limitation of this narrative review.

### 2.3. Eligibility Criteria

Studies were eligible for inclusion if they met the following criteria (see Table 1):

- Population: Children aged 5–12 years at baseline, with follow-up assessments extending into adolescence (13–18 years).
- Exposure: Studies assessed multidimensional physical literacy or one or more recognized domains of physical literacy (e.g., physical competence, motivation, confidence, knowledge, or understanding) using validated instruments or established conceptual frameworks.
- Outcome: Longitudinal assessment of PA participation or movement behaviors, with at least one follow-up assessment occurring during adolescence.
- Study design: Longitudinal cohort, prospective or follow-up studies. Systematic reviews and meta-analyses were retained as secondary evidence only when they reported longitudinal evidence relevant to the review question.
- Publication characteristics: Peer-reviewed articles published in English.

Studies were excluded if they:

- Employed exclusively cross-sectional designs without a longitudinal component;
- Focused solely on clinical populations where findings were unlikely to be generalizable to typically developing children and adolescents;
- Did not assess physical literacy or one or more recognized domains of physical literacy;
- Were conference abstracts, dissertations, editorials, commentaries, or other forms of grey literature.

Systematic reviews and meta-analyses were used as secondary evidence and were not treated as primary studies. Studies that did not meet the population, temporal, or outcome criteria were excluded from the primary evidence synthesis. During synthesis, formally included primary studies were clearly distinguished from contextual or supporting literature (see Table 2 and Table 3).

### 2.4. Study Selection

Titles and abstracts were independently screened by two reviewers against the predefined eligibility criteria. Potentially eligible studies subsequently underwent full-text assessment. Any disagreements regarding study eligibility were resolved through discussion and, where necessary, consultation with a third reviewer until consensus was reached.

As this process was undertaken as a narrative review rather than a systematic review, detailed screening records were not retained during the review process. The databases searched, principal search concepts, eligibility criteria, study selection procedures, and synthesis approach are reported to enhance transparency; however, the original database-specific search strings, exact search dates, record counts, and detailed reasons for full-text exclusion were not retained and therefore cannot be reconstructed reliably. Because these records are unavailable, the identification and selection of studies cannot be independently verified or fully reproduced. This limitation is therefore given substantial weight when interpreting the very small primary evidence base and the confidence that can be placed in the review findings. Reporting guidance for systematic reviews, including the Preferred Reporting Items for Systematic Reviews and Meta-Analyses (PRISMA), provides a useful contrast for interpreting the reporting limitations of this narrative review [19,20].

### 2.5. Data Extraction and Narrative Synthesis

Data extracted from eligible studies included study design, participant characteristics, physical literacy construct(s) assessed, PA outcomes, follow-up duration, principal findings, and identified mediating or moderating factors. To minimize overinterpretation of the available evidence, primary longitudinal studies, systematic reviews, and meta-analyses were considered separately during the synthesis. Attention was given to distinguishing studies assessing multidimensional physical literacy from those examining individual domains, especially motor competence, recognizing that these constructs should not be interpreted as equivalent.

Given the substantial conceptual and methodological heterogeneity across studies, a narrative synthesis was undertaken.

### 2.6. Scope of the Review

This narrative review focused on typically developing children and adolescents and examined longitudinal evidence relating childhood physical literacy, or its constituent domains, to subsequent PA participation during adolescence. Studies from diverse geographical and cultural settings were included to maximize the breadth of available evidence while recognizing differences in educational systems, cultural contexts, and approaches to measuring physical literacy.

## 3. Results

Clark et al. [21] was reassessed against the prespecified temporal outcome criterion and was not retained as a primary longitudinal study. The study followed 2087 Canadian students across Grades 5 to 7. The published article reports assessment points by grade rather than providing exact participant ages at each wave; importantly, its PA and sedentary-behavior trajectory analyses were conducted across Grades 5 and 6, while the Grade 7 outcomes were BMI and self-concept rather than PA. The PA outcomes therefore do not meet the review’s requirement for an adolescent PA assessment (13–18 years). The study is retained as contextual evidence of the association between multidimensional physical literacy and PA in late childhood/early adolescence, but it is not included in the primary synthesis, Table 2 and Table 3. Caldwell et al. [22] was also retained as contextual evidence because its temporal direction was reversed relative to the review question; preschool physical activity trajectories were examined as predictors of later school-age physical literacy, rather than childhood physical literacy being examined as a predictor of subsequent physical activity.

Lima et al. [17] examined motor competence across seven years, with assessments at mean ages 6.75, 9.59 and 13.35 years. Using accelerometry and structural equation modelling, motor competence and vigorous PA demonstrated reciprocal longitudinal associations (motor competence → vigorous PA β = 0.14; vigorous PA → motor competence β = 0.18), with cardiorespiratory fitness partially mediating the associations. This study provides evidence for the longitudinal importance of motor competence, but not for multidimensional physical literacy.

Jaakkola et al. [18] examined perceived physical competence towards PA and later PA across a six-year period. Among 333 Finnish students with a mean baseline age of 12.41 years, perceived physical competence was assessed in Grade 7 and self-reported PA was assessed in Grade 12. Perceived physical competence significantly predicted total METs, moderate PA and vigorous PA six years later (β = 0.28, 0.18 and 0.29, respectively), after accounting for relevant covariates. The findings support the potential relevance of perceived competence as one constituent domain of physical literacy, although the baseline cohort was already at the transition into adolescence and the exposure was domain-specific rather than multidimensional physical literacy.

Burton et al. [14] reported pooled associations between motor competence and PA (r = 0.20–0.26) across adolescent studies. The review included 61 studies overall and highlighted the lack of longitudinal observations as an important limitation. Accordingly, these pooled estimates are treated in the present review as contextual secondary evidence rather than as estimates of longitudinal associations, and motor competence is not treated as synonymous with multidimensional physical literacy. Together, these contextual findings highlight the need for longitudinal evidence to clarify temporal associations between physical literacy and subsequent PA.

Psychosocial, cognitive and academic outcomes were not primary outcomes of this review and were therefore not included in the main results synthesis, except where they directly informed interpretation of the relationship between physical literacy and adolescent PA. Related literature has examined associations between PA and academic achievement, executive function, mental health, and adolescent physical literacy and wellbeing [23,24,25,26,27,28,29].

Across the primary longitudinal evidence, effect estimates were generally modest. Simple correlations, adjusted longitudinal associations, and trajectory-based estimates were not treated as directly equivalent. Interpretation considered effect magnitude, confounder adjustment, measurement approach, follow-up duration, and study design. The small number of studies directly assessing multidimensional physical literacy limits confidence in generalizing findings from individual domains to the broader construct.

## 4. Discussion

This narrative review identified a small longitudinal evidence base examining early physical literacy and subsequent adolescent PA. Two primary studies met the prespecified eligibility criteria. Overall, the findings indicate positive associations between early physical literacy or its constituent domains and later PA, but the magnitude of associations was generally modest and direct evidence for multidimensional physical literacy remains limited.

Clark et al. [21] provided longitudinal evidence involving multidimensional physical literacy, but it did not meet the present review’s temporal outcome criterion because PA and sedentary-behavior trajectories were assessed in Grades 5–6 rather than during adolescence; the Grade 7 outcomes were BMI and self-concept. It is therefore considered contextual rather than primary evidence. In contrast, Lima et al. [17] and Jaakkola et al. [18] examined motor or perceived physical competence, respectively. These findings support the potential longitudinal relevance of individual physical literacy domains but should not be interpreted as equivalent evidence for the multidimensional construct. Cross-sectional studies of adolescent physical literacy and related outcomes were considered as contextual rather than longitudinal evidence [28,29,30]. Caldwell et al. [22] was considered contextual rather than primary evidence because it examined the reverse temporal pathway, with preschool physical activity trajectories predicting later school-age physical literacy rather than childhood physical literacy predicting subsequent physical activity.

Within the small primary evidence base, motor competence and perceived physical competence were positively associated with subsequent PA. Lima et al. [17] demonstrated reciprocal associations between motor competence and vigorous PA across childhood and early adolescence. However, the reciprocal nature of this relationship means that temporal ordering is bidirectional, limiting causal interpretation. This cautious interpretation is consistent with Barnett et al. [13], which characterized the broader longitudinal evidence for the motor competence–PA pathway as indeterminate.

Potential mechanisms included perceived competence, confidence, motivation and cardiorespiratory fitness. However, relatively few eligible primary longitudinal studies directly tested mediation or moderation, and the available evidence was heterogeneous. The contextual literature, including studies examining broader psychosocial or environmental factors, was considered only to assist interpretation and was not treated as direct evidence for the primary review question. The contextual literature concerning internalizing difficulties in children and young people also supports careful consideration of psychosocial pathways [25,26,27,31,32]. Studies examining diversified early-childhood activity and later physical literacy/wellbeing provide contextual support for considering reciprocal and psychosocial pathways [26,27].

The evidence base has several important limitations. Studies varied in physical literacy constructs, measurement instruments, PA assessment, follow-up duration and analytical methods. Objective PA measurement was available in some studies but not consistently. Formal risk-of-bias assessment was not undertaken; instead, study-level strengths and limitations were considered narratively, including attrition, confounding, measurement approaches, missing data and temporal sequencing. Consequently, statements about the strength, consistency or quality of the evidence should be interpreted cautiously and should not be taken to represent a formal methodological quality judgement.

Most importantly, only a very small number of longitudinal studies met the applied eligibility criteria, and none directly assessed multidimensional physical literacy with a subsequent adolescent PA outcome. Evidence from motor competence and perceived competence therefore cannot be generalized to the whole construct. In addition, the conceptual definition of physical literacy used in this review includes engagement in physical activity as a behavioral component, raising the possibility of construct overlap when physical literacy instruments incorporate PA participation. In the primary studies retained here, however, the exposure measures did not directly incorporate the same PA outcome: Lima et al. assessed motor competence using the KTK, while Jaakkola et al. assessed perceived physical competence using the sport competence dimension of the Physical Self-Perception Profile. The broader literature also includes evidence on physical fitness tracking and school-based physical literacy promotion, which is considered here only as contextual evidence [24,33,34].

Taken together, the findings indicate that, within the limited set of primary longitudinal studies identified, motor competence and perceived physical competence showed positive associations with subsequent PA. However, the overall evidence remains limited and heterogeneous, and no primary study meeting the applied criteria directly examined multidimensional physical literacy as an exposure with an adolescent PA outcome. The evidence is not sufficient to establish that developing multidimensional physical literacy causes more favorable adolescent PA trajectories. Future longitudinal studies should therefore prioritize comprehensive multidimensional assessments, objective PA measures, longer follow-up and examination of mediating and contextual factors.

## 5. Implications for Practice, Policy and Future Research

The findings have implications for education and public health, but these should be interpreted in proportion to the limited direct longitudinal evidence. Schools provide an important setting for diverse, enjoyable and inclusive movement experiences that may support physical literacy. However, the evidence does not establish that physical literacy development alone will produce sustained increases in adolescent PA. Practice and policy should therefore avoid causal assumptions and consider physical literacy alongside the multiple individual, social and environmental factors that influence PA. Evidence from physical literacy intervention and school-based literature provides additional context for these practice considerations [24,35].

Policy and practice should support equitable opportunities for children to develop movement competence, confidence, motivation and knowledge while recognizing that PA trajectories are influenced by multiple individual and environmental factors.

Key research priorities include validated multidimensional physical literacy measures, objective PA assessment, longer follow-up into late adolescence and adulthood, and investigation of mediating and moderating factors such as motivation, confidence, family support, school environments and socioeconomic influences.

## 6. Strengths and Limitations

This narrative review specifically examined longitudinal evidence relating early physical literacy to adolescent PA. It used predefined eligibility criteria and independent screening procedures and distinguished multidimensional physical literacy from individual domains, particularly motor competence. The review also separated primary longitudinal evidence from secondary review evidence.

Several limitations should be acknowledged. As a narrative review, detailed screening records were not retained, and the exact database-specific search strings, final search date, record counts, and detailed reasons for full-text exclusion are unavailable. Consequently, the search cannot be reproduced in full, and the identification and selection of the very small primary evidence base cannot be independently verified. Formal risk-of-bias assessment was also not undertaken; study-level strengths and limitations were instead considered narratively. Accordingly, terms such as strength, consistency and quality of evidence are used cautiously and should not be interpreted as formal risk-of-bias judgements. The exclusion of non-English publications may have introduced language bias, and the small number of eligible studies, together with the absence of direct multidimensional physical literacy evidence, limits confidence in conclusions about the complete construct. Potential construct overlap between physical literacy and PA is also a limitation of the broader conceptual framework, although the primary exposure measures retained in this review did not directly incorporate the subsequent PA outcome.

## 7. Conclusions

This narrative review synthesized longitudinal evidence examining early physical literacy and subsequent PA during adolescence. Within the limited set of primary longitudinal studies identified, motor competence and perceived physical competence showed positive associations with subsequent PA. However, the overall evidence remains limited and heterogeneous, and direct evidence for multidimensional physical literacy remains insufficient. Associations were generally modest, and substantial heterogeneity in physical literacy and PA measurement limits direct comparison and causal inference. Future research should prioritize comprehensive multidimensional assessment, objective PA measures, longer follow-up and investigation of mechanisms and contextual factors.

## Figures and Tables

**Table 1:** Inclusion and Exclusion Criteria.

Criterion	Inclusion Criteria	Exclusion Criteria
Population	Children in childhood or the transition to early adolescence at baseline (approximately 5–12 years), with follow-up into adolescence (13–18 years)	Clinical or highly specialized populations where findings were not generalizable to typically developing children and adolescents
Exposure	Physical literacy or one or more recognized domains (e.g., physical competence, motivation, confidence, knowledge, understanding) assessed using validated measures or recognized frameworks	Studies not assessing physical literacy or its recognized domains
Outcome	Longitudinal assessment of PA or movement behaviours, with at least one follow-up PA assessment occurring during adolescence (13–18 years)	Studies without longitudinal PA outcomes
Study design	Longitudinal cohort, prospective or follow-up studies meeting the population and temporal outcome criteria; systematic reviews/meta-analyses used as secondary evidence where relevant	Cross-sectional studies only; studies without longitudinal PA outcomes; conference abstracts; editorials; commentaries; dissertations; grey literature
Language	English	Non-English publications
Publication period	Published from 2000 onwards	Published prior to 2000

**Table 2:** Characteristics of primary longitudinal studies meeting the eligibility criteria.

Study	Country	N	Baseline Age	Follow-Up	PL/Domain Measure	PA Assessment	Covariates/Analysis	Main Effect Estimate	Risk/Limitations
Lima et al. (2017) [17]	Denmark	696 baseline; 513 at age 13.35	6.75 ± 0.37 y	7 years; age 13.35 y	Motor competence (KTK)	Accelerometry	Structural equation modelling; cardiorespiratory fitness examined as mediator	MC → VPA β = 0.14; VPA → MC β = 0.18; VO_2_peak mediated both pathways	Motor competence only; attrition across waves
Jaakkola et al. (2016) [18]	Finland	333	12.41 ± 0.27 y	6 years; Grade 12	Perceived physical competence/sport competence domain	Self-reported IPAQ-SF	Sex; previous PA and BMI controlled	Perceived competence predicted total METs β = 0.28, MPA β = 0.18, VPA β = 0.29	Baseline at the transition into adolescence; self-reported PA; domain-level evidence only

**Table 3:** Characteristics of primary longitudinal studies meeting the applied eligibility criteria.

Source	Evidence Type	Evidence Base	Key Finding	Interpretive Limitation
Barnett et al. (2021) [13]	Systematic review	Longitudinal evidence on motor competence and health	Indeterminate evidence for the motor competence → physical activity pathway	Heterogeneous studies; not focused specifically on multidimensional PL
Burton et al. (2023) [14]	Systematic review and meta-analysis	61 studies; 10 longitudinal	Pooled MC–PA associations r = 0.20–0.26; evidence base predominantly cross-sectional, with limited longitudinal observations	Modest associations; heterogeneous designs and measures; longitudinal evidence limited

Key: MC: Motor Competence; PA: Physical Activity; VPA: Vigorous Physical Activity; PL: Physical Literacy. The primary evidence synthesis included three longitudinal studies; secondary reviews were considered separately.

## Data Availability

No new data were created or analyzed in this study. Data sharing is not applicable to this article.
